# Mortality attributable to influenza-like illness, gastro-enteritis and pneumonia, results from the Dutch Sentinel Network for Surveillance of Infectious Disease (SNIV)

**DOI:** 10.1186/1753-6561-5-S6-P166

**Published:** 2011-06-29

**Authors:** M-J Veldman-Ariesen, L van Asten, AP Haenen, M Kretzschmar, BH van Benthem, SNIV study group

**Affiliations:** 1 Epidemiology and Surveillance Unit, Dutch National Institute for Public Health and the Environment (RIVM), Centre for Infectious Disease Control, Bilthoven, Netherlands

## Introduction / objectives

Elderly and especially nursing home residents are at increased risk of infectious diseases due the high prevalence of underlying chronic illnesses, age-related immunesenesence and the close(d) environment typical for this population. The impact of the occurence of infectious diseases on mortality is subject of this study.

## Methods

In the Sentinel Surveillance Network on Infectious diseases in nursing homes (SNIV), elderly care physicians and/or nurse practitioners report weekly numbers of 1) mortality and 2) infectious diseases on the basis of clinical definition. On these weekly time series (mid 2008- beginning of 2011) we used Poisson regression models (which included linear and periodic components) to characterize the association of total death counts with trends in influenza-like illness (ILI), gastro-enteritis (GE), and probable pneumonia.

## Results

In total 35 nursing homes with a total of 4516 residents participated during part of the 128 week study period. The incidence of mortality and infectious illnesses displayed seasonal peaks in winter. In total 2324 residents died. Per 1000 residents, each reported case of pneumonia was associated with 3.4 (95%CI:1.7-5.2) deaths occurring in the same week and 2.2 (95%CI:0.5-3.9) occurring 2 weeks later. Each reported GE case was associated with 1.1 (95%CI:0.4-1.7) deaths one week later. Together with these infections, the linear and periodic terms were no longer significant and were thus excluded from the final model. Of all deaths, our model attributed 745 (32%) to pneumonia and 172 (7%) to GE.

## Conclusion

Probable pneumonia and gastro-enteritis were significantly associated with overall mortality, while influenza-like illness was not.

## Disclosure of interest

None declared.

